# Strength in Numbers: The WWARN Case Study of Purpose-Driven Data Sharing

**DOI:** 10.4269/ajtmh.18-0649

**Published:** 2018-10-22

**Authors:** Georgina S. Humphreys, Halidou Tinto, Karen I. Barnes

**Affiliations:** 1 WorldWide Antimalarial Resistance Network (WWARN), University of Oxford, Oxford, United Kingdom;; 2 Centre for Tropical Medicine, Nuffield Department of Clinical Medicine, University of Oxford, Oxford, United Kingdom;; 3 Institut de Recherche en Sciences de la Santé-Clinical Research Unit of Nanoro (IRSS-CRUN), Ouagadougou, Burkina Faso;; 4 Division of Clinical Pharmacology, Department of Medicine, University of Cape Town, Cape Town, South Africa;; 5 WorldWide Antimalarial Resistance Network (WWARN) Pharmacology and Southern African Regional Hub, University of Cape Town, Office K27-24 Old Main Building Groote Schuur Hospital, Anzio Road, Observatory, 7925, Cape Town, South Africa

## Abstract

Data are the basis for all scientific output. The sharing of data supporting that output is an important aspect of scientific communication, and is increasingly required by funders and publishers. Yet, academic advancement seldom recognizes or rewards data sharing. This article argues that although mandating data sharing will increase the amount of data available, this will not necessarily enable or encourage the secondary analyses needed to achieve its purported public good. We, therefore, need to build models that maximize the efficiency of processes for data collation and curation, and genuinely reward those engaged in data sharing and reuse. The WorldWide Antimalarial Resistance Network has 10 years of experience as a data platform, and its study group approach provides an example of how some of the challenges in equitable and impactful data-sharing and secondary use can be addressed, with a focus on the priorities of researchers in resource-limited settings.

Data sharing is important, and increasingly mandated by funders and publishers, which will inevitably result in an increased number of datasets being shared. However, more now needs to be done to ensure that sharing these data results in the expected benefits of increased scientific productivity, efficiency, and public health impact. In the context of data collected in low- and middle-income countries, concerns have been raised regarding inequitable opportunities to engage in secondary use of data between researchers in well-resourced and resource-limited settings. This is of particular importance for research in poverty-related diseases, such as malaria, neglected tropical diseases, and emerging infections such as Ebola. In such fields, differences in technical and financial capacity between primary data producers and secondary data users are often large. Although primary data originate in resource-limited settings, it is in high-income settings where resources are more readily available for conducting secondary analyses.^1,2^

Meta-analysis based on aggregated information extracted from publications remains the most widely used method to synthesize evidence to guide policy recommendations. However, results based on published aggregated data alone may be limited and potentially misleading because of publication bias and heterogeneity in methodologies.^3^ Data are inconsistently reported, with key information often missing.^4^ Heterogeneity of methodologies often restricts the feasibility of the meta-analysis approach.^5^ For example, an aggregated data meta-analysis on early parasitological response following treatment with artemisinin-containing regimens found that the proportion of patients remaining parasitemic at 24, 48, and 72 hours was reported in less than half of the relevant publications, highlighting the need for individual patient data (IPD) meta-analyses.^6^

Individual patient data meta-analysis is an increasingly recognized alternative method to evidence synthesis that enables standardization of outcomes across studies, and may improve control of bias.^3,7,8^ Unlike most aggregated meta-analyses, the IPD approach is often applied beyond randomized controlled trials (RCTs). This allows for inclusion of other study designs that may be more representative of the normal context of use. Individual patient data also gives greater statistical power and analytical flexibility—for example, the ability to study drug efficacy for a particular subgroup of patients who are an important target population, but usually underrepresented in individual clinical trials. Huang et al.^9^ compared results from aggregated meta-analyses and IPD meta-analyses and found that among the 204 paired studies, 91.7% agreed in the overall effect, but the IPD meta-analyses enabled seven-times more subgroup analyses and identified 14 times more potential interactions.

Notwithstanding the advantages of the IPD approach, using IPD from clinical trials can raise ethical, logistical, and methodological challenges. These may include reuse for purposes not originally intended when consent was agreed, inadequate recognition of the primary generators of the data, and regulatory issues such as national policies that limit data sharing. The challenges of collating, curating, and analyzing IPD are significant, and certainly more complex and resource-consuming than for aggregate data. This is reflected in the relative scarcity of IPD meta-analyses in the literature; in a survey of corresponding authors of meta-analyses of RCTs in general medicine, only 4.2% included IPD.^10^

In other health disciplines, such as genomics, the culture of data sharing and reuse is better established, with clear evidence of a citation benefit for studies with a publicly available dataset.^11^ However, similar evidence from clinical and epidemiological studies is limited. Although there are some recent examples for controlled access data sharing and analysis of clinical trials data, these largely focus on data collected in resource-rich settings where data management standards make it easier to share, curate, and combine datasets.^12,13^

Individual patient data meta-analyses are particularly important in the field of poverty-related infectious diseases that have relatively low levels of research funding, and so relatively few data are available. Thus, the potential for new findings from bringing these sparse data together is much higher. Reviews of the published literature on malaria, schistosomiasis, soil-transmitted helminths, and visceral leishmaniasis highlighted the importance of IPD meta-analyses to not only increase statistical power, but also to study specific subpopulations whose outcomes are often hidden in aggregated data,^14,15^ thus ensuring that the full public health benefit of these studies is realized.

There are a number of ways to ensure or encourage sharing: to pay for access to data, to offer the incentive of co-authorship of the secondary use publication, and, most often, funders and publishers mandating data sharing. However, the current academic culture often runs counter to data sharing, as it promotes individual achievement and encourages academics to be proprietorial. Enforcing data sharing, without incentives, may merely compel researchers to meet only a minimum set of requirements, and not fully engage in sharing all information needed to make meaningful secondary use possible.^16^

More creative models of data sharing and secondary analysis are needed. Although there has been much discussion about potential benefits and concerns around data sharing, particularly for those researchers based in low- and middle-income countries,^1,16^ there are very few examples of successful models for purpose-driven data sharing and secondary analysis. To address this gap, the WorldWide Antimalarial Resistance Network (WWARN) launched its first study group in 2011, which brought data contributors together to conduct purpose-driven, equitable IPD meta-analyses with support from core WWARN staff. The study groups were driven by a shared interest in making the best use of available data for answering a specific question, and the commitment to encourage full involvement and ensure recognition for data contributors. Study group membership is open to anyone with relevant data. This approach was designed not only to ensure appropriate recognition of those researchers who invest significant resources in collecting the data, but also to benefit from the participants’ scientific expertise and detailed understanding of their data and the context in which it was collected. Depending on each study group member’s level of engagement, the members are authors, collaborators, or personally acknowledged in resulting publications. Much has been learnt through WWARN’s pioneering efforts; this has been independently evaluated in a case study by Pisani and Botchway.^17^

WorldWide Antimalarial Resistance Network increases efficiency and reduces data loss by standardizing and storing datasets for multiple reuse without needing repeated data curation. By combining IPD from studies conducted by many different groups and in many different countries and settings, it has been possible to observe patterns in malaria treatment efficacy that would not have been discernible in an individual study. The nine publications generated by study groups^18–26^ up to December 2017 have included a total of 244 authors, with contributions from 189 separate study datasets shared through the WWARN platform. More than 60% (114) of the studies used in these nine pooled analyses have been included in more than one study group, with 36 (19%) study datasets used in four or more analyses.

An example of the impact of WWARN’s IPD meta-analyses is a report that included more than 7,000 patients given dihydroartemisinin–piperaquine for the treatment of uncomplicated malaria from 26 studies.^21^ The pooling of these data showed generally high cure rates, with only 136 treatment failures, but that young children were three times more likely to fail treatment than older patients because they were suboptimally dosed. These findings were subsequently supported by pharmacometric models,^27^ and ultimately contributed to dosage recommendation changes in the WHO treatment guidelines.^28^ Another example is the Parasite Clearance study group^23^ that pooled IPD from more than 6,900 patients enrolled in 24 studies with frequent parasite counts. This pooled analysis was used to validate a tool developed to standardize estimates of the parasite clearance rates that has been cited by more than 100 subsequent peer-reviewed publications.^29^ WorldWide Antimalarial Resistance Network is building on this experience to understand how best to encourage more malaria endemic country–based researchers to lead study groups, including providing more tools, infrastructure, and technical support to enable them to answer the questions most relevant to them. We should continue to innovate by experimenting with different data sharing models, particularly as data sharing practices develop and mechanisms for incentivizing and recognizing data sharing are established.

To realize the full potential of shared data, it is imperative that funders work together with researchers to launch funding calls specifically targeted at secondary analysis. In addition, long-term infrastructure support is required to efficiently curate and store reusable data. Given the challenges of data collection and collation, we have a responsibility to retain its value and maximize its impact. If we can create trusted environments, such as WWARN, where skills and knowledge are shared, those contributing are recognized and inefficiencies are reduced, then the benefits of data sharing will be enhanced and more evenly distributed. By embracing a purpose-driven equitable approach to data sharing, the potential to learn more from the data already generated by staff and patients around the world will be unlocked.
